# Analysis of predictive factors for immunosuppressive response in anti-phospholipase A2 receptor antibody positive membranous nephropathy

**DOI:** 10.1186/s12882-018-1160-6

**Published:** 2018-12-12

**Authors:** Chao Li, Hang Li, Yu-bing Wen, Xue-mei Li, Xue-wang Li

**Affiliations:** Division of Nephrology, Peking Union Medical College Hospital, Chinese Academy of Medical Sciences and Peking Union Medical College, No. 1, Shuaifuyan, Wangfujing St, Beijing, 100730 China

**Keywords:** Glomerulonephritis, membranous, Proteinuria, Receptors, phospholipase A2, Autoantibodies

## Abstract

**Background:**

Serum anti-phospholipase A2 receptor (PLA2R) antibody was correlated with disease activity of membranous nephropathy(MN). The predictive value of antibody titer changes on immunosuppressive response remains unknown. We investigated predictive value of dynamic change of anti-PLA2R antibody and 24-h urine protein (24hUP) for clinical response of MN.

**Methods:**

This was a retrospective cohort study including 47 Chinese MN patients with positive anti-PLA2R antibody in a tertiary referral hospital between January 2012 and March 2014. Patients received cyclophosphamide (CTX, *n* = 23), or cyclosporine (CYA, *n* = 24) regimen, respectively. We monitored serum anti-PLA2R titer and 24hUP at one, three and six-month follow-up.

**Results:**

At baseline, total patients were 42 ± 14 years old with 29/18 male/female ratio. The median 24hUP was 5.80(3.56,9.41) g/d. The median baseline anti-PLA2R antibody titer was 66.4(31.9, 188.0) RU/mL. Baseline 24hUP and eGFR between subgroups were not significantly different. The differences of relative reduction between antibody titer and 24hUP at one month were statistically significant (CTX group 94.2% vs. 46.8%, *P* < 0.001; CYA group 54.6% vs. 4.6%, *P* = 0.04). Only in CTX group, the relative reduction of 24hUP at one month was correlated with composite remission at six-month(*P* = 0.03). Area under the curve of 24hUP relative reduction in CTX group at one-month for predicting composite remission at six months was 0.85(95%CI 0.65~1.05, *P* = 0.04). The cutoff value of one-month’s 24hUP relative reduction for predicting six-month’s composite remission in CTX group was 15.3%, with high sensitivity (83.3%) and specificity (100%).

**Conclusions:**

Compared with relative reduction of antibody titer, relative reduction of 24hUP at one-month follow-up in CTX group had a better predictive value for six-month’s composite remission.

## Background

Idiopathic membranous nephropathy (IMN) is an organ-specific autoimmune disease. The discovery of autoantibody against podocyte M-type phospholipase A 2 receptor (PLA2R) in 2009 highlighted immune-mediated pathogenesis of MN [1]. Serum anti-PLA2R antibody was not only useful in diagnosing IMN [2, 3] (70–80% in sensitivity, and more than 95% in specificity), but also correlated with disease activity [4, 5]. Several studies indicated that persistent high antibody levels were associated with a reduced probability of remission either spontaneously or after immunosuppressive therapy [4, 6]. Fervenza et al. suggested an individualized serology-based approach to MN [7]. However, the predictive value of antibody titer changes after treatment on clinical outcome was not well defined. There was no consensus on details of immunosuppressive treatment modulation based on autoantibody monitoring, such as frequency of monitoring autoantibody, when to adjust drug dose based on antibody titer change. In our study, we monitored the anti-PLA2R antibody titer dynamically in IMN patients treated with either cyclophosphamide plus corticosteroid or cyclosporine plus corticosteroid, in order to answer whether the predictive value of antibody titer change for clinical response was better than that of proteinuria change in early stage after treatment.

## Methods

### Study population

This was a retrospective cohort study of Chinese IMN patients hospitalized in Peking Union Medical College Hospital (PUMCH), a tertiary referral hospital, between January 2012 and March 2014. Inclusion criteria were as followings: no less than 18 years old when renal biopsy; newly diagnosis of MN confirmed by renal biopsy; positive serum anti-PLA2R antibody titer at diagnosis; no spontaneous remission after conservative treatment (ACE inhibitors/angiotensin II receptor blocker, statin and diuretics) for at least three months. Spontaneous remission was defined as proteinuria < 3.5 g/24 h and at least 50% reduction from the time of initiating conservative treatment. Exclusion criteria were as followings: presence of any secondary cause of MN (drug/toxin exposure, viral infection, autoimmune disease or malignancy); any use of corticosteroid or immunosuppressive medication within six months before inclusion to study. Totally 47 patients who met all the criteria were included.

The included patients received either cyclophosphamide plus corticosteroid based on modified Ponticelli protocol [8] or cyclosporine (3.5 mg/kg per day as initial dose and adjusted to maintain cyclosporine trough level of 125-175 ng/mL) plus prednisone (0.15 mg/kg/d). The regimen choice was decided by renal physicians with consideration of patient’s preference and drug potential side effect. The study was approved by PUMCH ethical committees (No. S-K014) and informed consents was obtained from included patients for blood sampling and renal biopsy.

The clinical data collected and analyzed as followings: age at diagnosis, gender, body mass index (BMI), blood pressure. The laboratory assessment included serum creatinine (Scr), serum albumin, and 24-h urine protein(24hUP). Estimated glomerular filtration rate (eGFR) was calculated by Chronic Kidney Disease-Epidemiology Collaboration (CKD-EPI) equation.

The renal biopsy specimens of all the patients were examined by light microscopy, immunofluorescence and electron microscopy.

The included patients were followed up regularly at one, three and six months in clinic. After that, renal physicians providing medical care to patients decided the follow-up frequencies by themselves. The last follow-up date of our study was on June 30th, 2017.

Complete remission (CR) was defined as proteinuria≤0.3 g/d, serum albumin> 35 g/L with stable eGFR. Partial remission (PR) was defined as a reduction > 50% of the baseline proteinuria (with a lower value of < 3.5 g/d) with stable eGFR. A stable GFR was defined as a GFR remaining unchanged or declines by < 15% during follow-up. Composite remission included both complete remission and partial remission. No response (NR) was defined as absence of complete or partial remission at six months after immunosuppressive treatment initiation. Relapse was defined as recurrence of proteinuria> 1 g/d after achieving complete remission.

The primary outcome was probability of composite remission at six-month follow-up. The secondary outcome was clinical response at the end of follow-up.

The serum samples collected before and during immunosuppressive treatment (one, three and six-month’s follow-up) were stored at − 80 °C for measuring anti-PLA2R antibody titer. Anti-PLA2R antibody titer was measured by ELISA method (Euroimmun China, Inc). According to the indication of the manufacturer, a negative antibody titer was defined as an anti-PLA2R antibody titer <14 RU/ml.

### Statistical analyses

Baseline data and outcome were expressed as percentages for categorical variables, means and standard deviations (SD) for normally distributed continuous variables, or median and quartiles (P_25_, P_75_) for non-normally distributed continuous variables. Differences in categorical variables were analyzed using the Chi-squared test or Fisher’s exact test. Differences in continuous variables were analyzed using Student *t*-test for normally distributed variables or the Wilcoxon test for non-normally distributed variables. We used rank correlation analysis between relative reduction of antibody titer or proteinuria at one or three months and composite remission at six-month follow-up. We analyzed the predictive value of the relative reduction in antibody titer and proteinuria at early stage by using receiver operating characteristic curve (ROC) curves. All patients who were not in remission at one month were included for the calculation of the predictive value of the reduction in antibody titer and 24hUP at one month on clinical response at six months. For calculation of the predictive value of the relative reduction in antibody titer and 24hUP at three months on clinical response at six months, patients who were in complete or partial remission at three months were excluded for calculation. The slope of reduction in antibody titer was calculated during the follow-up and expressed as RU/ml/month.

A two-sided *P* value of < 0.05 was considered statistically significant. The SPSS version 19.0 statistical software package (SPSS Inc.) and SigmaPlot version 13.0 (Systat Software Inc.) were used for the analyses.

## Results

### Baseline characteristics

At baseline, 47 patients were 43 ± 14 years old with 29/18 male/female ratio. Mean serum albumin was 27.1 ± 5.6 g/L, while median 24hUP was 5.80 (3.56, 9.41) g/d. Average serum creatinine was 74.9 ± 22.0 μmol/L, corresponding eGFR (CKD-EPI) 109.1 ± 26.1 mL/min per 1.73m^2^. The baseline serum anti-PLA2R antibody titer was 66.4 (31.9, 188.0) RU/mL. The mean systolic and diastolic blood pressure were 127 ± 12 mmHg and 78 ± 10 mmHg, respectively. Table 1 summarized the clinical and laboratory baseline values of IMN patients in two groups. The differences of above mentioned baseline data between CTX and CYA groups were not statistically significant. Cyclosporine trough levels in CYA group were 137.7 ± 31.5 ng/mL.

**Table 1:** Comparison of baseline clinical and laboratory characteristics between CTX and CYA groups

Characteristics	CTX group (*n* = 23)	CYA group (*n* = 24)	*P* value
Men, *n* (%)	16 (69.6)	13 (54.2)	0.28
Age (yrs), mean ± SD	43 ± 12	42 ± 15	0.72
Body mass index (kg/m^2^), M (P_25_, P_75_)	25 (23, 29)	25 (23, 27)	0.87
Serum albumin (g/L), mean ± SD	26.1 ± 5.3	28.0 ± 5.8	0.25
24hUP (g/d), M (P_25_, P_75_)	8.28 (3.63, 10.98)	4.73 (3.38, 8.60)	0.14
Nephrotic syndrome, *n* (%)	14 (60.9)	16 (66.7)	0.68
Scr (μmol/L), mean ± SD	78.7 ± 24.7	71.3 ± 19.0	0.26
eGFR (mL/min per 1.73m^2^), mean ± SD	103 ± 28	115 ± 24	0.15
Lymphocyte count (×10^9^/L), mean ± SD	2.51 ± 1.04	2.25 ± 0.81	0.32
Anti-PLA2R antibody titer (RU/ml), M (P_25_, P_75_)	66.4 (31.5, 192.4)	63.4 (33.6, 174.0)	0.69
Systolic pressure (mmHg), mean ± SD	127 ± 8	127 ± 14	0.995
Diastolic pressure (mmHg), mean ± SD	77 ± 11	78 ± 10	0.75

*CTX* cyclophosphamide, *CYA* cyclosporine A, *Scr* Serum creatinine, *eGFR* estimated glomerular filtration rate, *PLA2R* phospholipase A 2 receptor

### Changes of serum anti-PLA2R antibody titer after treatment

**Fig. 1:**
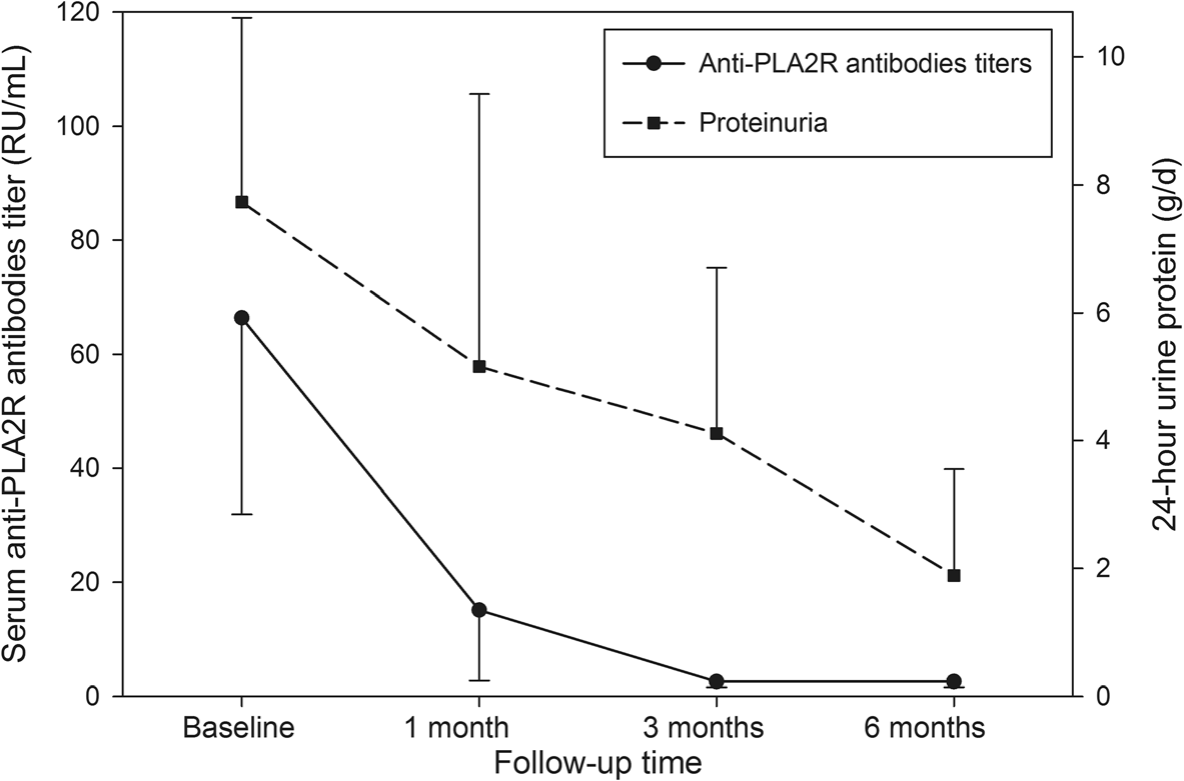
The dynamic change of serum anti-PLA2R antibody titer and 24hUP during follow-up in total population

Figure 1 described the declining trend of serum anti-PLA2R antibody and 24hUP during immunosuppressive treatment in total patients. Noticeably, the largest slope of antibody titer reduction [29.7(− 1.6, 104.3) RU/ml/month] occurred at one month after treatment. The evolution of serum anti-PLA2R antibody titer in two groups during six-month treatment period was summarized in Table 2. It showed that the patients in CTX group had significantly higher immunological response rate than those in CYA group at one month after treatment (median antibody titer in CTX group and CYA group, 3.6 RU/mL vs. 30.1 RU/mL, *P* = 0.04; seroconversion rate of antibody in CTX group and CYA group, 69.6% vs. 29.2%, *P* = 0.01). However, the above-mentioned difference between the two groups was not statistically significant after the third month treatment.

**Table 2:** Dynamic evolution of serum anti-PLA2R antibody titer in CTX and CYA groups during the six-month immunosuppressive treatment period

Characteristics	CTX group (*n* = 23)	CYA group (*n* = 24)	*P* value
Anti-PLA2R titer (RU/mL), M (P_25_, P_75_)			
One month	3.6 (1.6, 58.9)	30.1 (3.8, 130.7)	0.04
Three months	1.9 (1.5, 30.7)	3.9 (2.1, 43.1)	0.06
Six months	1.7 (1.3, 4.3)	2.9 (1.6, 36.4)	0.08
Seroconversion of Anti-PLA2R antibody, *n* (%)			
One month	16 (69.6)	7 (29.2)	0.01
Three months	17 (73.9)	14 (58.3)	0.26
Six months	18 (78.3)	16 (66.7)	0.37

*CTX* cyclophosphamide, *CYA* cyclosporine A, *PLA2R* phospholipase A 2 receptor

### Clinical response after treatment

The composite remission rates in total population at one and three months were 23.4% (11 PR patients without CR patients) and 38.3% (15 PR patients and three CR patients). At the end of follow-up, this remission rate increased to 80.9% (13 PR patients and 25 CR patients), whereas the no response rate and relapse rate were 8.5%(*n* = 4) and 10.6%(*n* = 5), respectively. The clinical responses of each group were summarized in Table 3. No significant differences in the composite remission rate were observed between the two groups, either at six-month follow-up or at study endpoint.

**Table 3:** Clinical response evaluation during six-month treatment period and follow-up endpoint in CTX and CYA group patients

Characteristics	CTX group (*n* = 23)	CYA group (*n* = 24)	*P* value
24hUP(g/d), M (P_25_, P_75_)			
One month	3.37 (2.15, 6.21)	5.22 (3.08, 10.43)	0.18
Three months	2.51 (1.28, 4.76)	4.79 (2.80, 7.22)	0.03
Six months	1.37 (0.28, 2.41)	2.26 (1.18, 4.79)	0.05
Endpoint	0.31 (0.12, 3.48)	0.38 (0.22, 1.82)	0.70
Scr (μmol/L), M(P_25_, P_75_)			
Six months	69 (62, 86)	77 (59, 85)	0.48
Endpoint	75 (62, 90)	72 (58, 79)	0.38
eGFR (ml/min per 1.73m^2^), mean ± SD			
Six months	97.6 ± 26.5	98.4 ± 28.6	0.93
Endpoint	97.1 ± 19.6	102.9 ± 30.7	0.44
Lymphocyte counts (×10^9^/L)			
One month	2.73 ± 1.40	3.18 ± 1.32	0.45
Three months	2.44 ± 1.11	3.51 ± 1.57	0.01
Six months	2.38 ± 1.29	3.37 ± 1.52	0.02
Endpoint	2.22 ± 1.07	2.49 ± 1.03	0.39
Response at six-month’s follow -up [*n* (%)]			
Complete remission (CR)	10 (43.5)	5 (20.8)	0.24
Partial remission (PR)	9 (39.1)	12 (50.0)	0.24
Composite remission (CR + PR)	19 (82.6)	17 (70.8)	0.34
No remission (NR)	4 (17.4)	7 (29.2)	0.34
Median follow-up time (months), M (P_25_, P_75_)	29 (23, 32)	29 (15, 34)	0.92
Response at follow-up endpoint, *n* (%)			
Complete remission (CR)	13 (56.5)	12 (50.0)	0.10
Partial remission (PR)	4 (17.4)	9 (37.5)	0.10
Composite remission (CR + PR)	17 (73.9)	21 (87.5)	0.29
No remission (NR)	3 (13.0)	1 (4.2)	0.29
Relapse	3 (13.0)	2 (8.3)	0.67

*CTX* cyclophosphamide, *CYA* cyclosporine A, *Scr* Serum creatinine, *eGFR* estimated glomerular filtration rate, *24hUP* 24-h urine protein

### Prediction value of antibody titer and 24hUP reduction at early stage for composite remission at six-month follow-up

We summarized the relative reduction of serum anti-PLA2R titer and 24hUP during six-month’s follow-up in two groups (Table 4). The relative reduction of 24hUP in CTX group was significantly higher than those in CYA group, at one, three and six months after treatment. However, the significant difference of relative reduction in antibody titer between two groups was only observed at one-month follow-up. Within each group, the relative reduction in antibody titer preceded the reduction in proteinuria and was statistically significant at one month (*P* < 0.001 in CTX group, and *P* = 0.04 in CYA group).

**Table 4:** The relative reduction of 24hUP and anti-PLA2R antibody titer at one, three or six–month’s follow-up in CTX and CYA group patients

Characteristics	CTX group (*n* = 23)	CYA group (*n* = 24)	*P* value
Relative reduction of 24hUP (%), M (P_25_, P_75_)			
One month	46.8 (7.2, 67.2)	4.6 (−57.2, 32.5)	< 0.01
Three months	66.9 (30.9, 78.7)	22.7 (−66.0, 46.6)	0.01
Six months	81.0 (68.0, 95.0)	58.5 (10.8, 79.5)	0.02
Relative reduction of anti-PLA2R titer (%), M (P_25_, P_75_)			
One month	94.2 (69.1, 97.3)	54.6 (−34.9, 94.7)	0.03
Three months	97.0 (84.2, 98.9)	92.3 (−15.5, 96.4)	0.07
Six months	97.0 (93.0, 99.0)	93.5 (79.3, 97.0)	0.22

*CTX* cyclophosphamide, *CYA* cyclosporine A, *SCr* Serum creatinine, *PLA2R* phospholipase A 2 receptor, *24hUP* 24-h urine protein

By Spearman rank correlation analysis, we did not find significant association between relative reduction of serum anti-PLA2R antibody at one or three months and composite remission at six-month follow-up, in either total population or any group. Only in CTX group, the relative reduction of 24hUP at one month was significantly correlated with composite remission at six-month follow-up (Coefficient = 0.53, *P* = 0.03).

By ROC analysis, area under the curve of 24hUP relative reduction in CTX group at one-month follow-up for predicting composite remission at six months was 0.85(95% CI 0.65~1.05, *P* = 0.04). The cutoff value of one-month’s 24hUP relative reduction for predicting six-month’s composite remission in CTX group was 15.3%, with sensitivity (83.3, 95% CI 51.6~97.9) and specificity (100, 95%CI 39.8~100.0).

## Discussion

The results of our study provided the following clinical relevant information. Firstly, our results coincided with the previous study that serological response preceded clinical response after immunosuppressive treatment [9, 10]. In our study, the maximum slope of antibody titer reduction occurred at one month, which was higher than that of proteinuria during the same period. Seroconversion of antibody occurred earlier than proteinuria remission at one month and the difference became insignificant at six months. All above observations were rationale given the proposed pathophysiology of IMN.

The second point of our study was the different dynamic evolution of antibody titer in two immunosuppressive groups, with no significant different clinical characteristics and antibody levels at baseline. CTX group had higher proteinuria than CYA group at baseline. Although the difference of proteinuria does not reach statistically significance, it could have been due to lack of power to detect the significance. CTX group had earlier immunological response than CYA group, as well as clinical response during six-month follow-up. The lymphocyte counts in CTX group were less than those in CYA group during follow-up. It was rational considering side effect of bone marrow suppression during CTX accumulation. However, both groups had similar composite remission rates at the end of follow-up, which was consistent with previous study results [11]. In addition, the relapse rate in CTX group was higher compared with that in CYA group, which was contrary to results of previous literature [12], although the difference was not significant. It could be associated with long term maintenance therapy with low dose cyclosporine commonly used in CYA group of our study, in order to prevent MN from relapse.

The novelty of our study was that we compared the predictive value for remission probability at six-month follow-up between relative reduction of anti-PLA2R antibody titer and 24hUP at early stage after treatment. Although antibody titer decreased more rapidly than proteinuria at early stage after treatment, it did not have statistical power as relative reduction of proteinuria for predicting the likelihood of composite remission at six-month. Our result was different from Spanish study [13], which showed statistically significant predictive value of relative reduction in anti-PLA2R antibody titer either in CTX group or tacrolimus combined with rituximab group. Several factors should be considered for the contrary results of two studies. Firstly, in our study, the significant data dispersion of anti-PLA2R antibody titer in both groups and 24hUP in CYA group could interfere their correlations with six month’s remissions. Secondly, the different baseline antibody levels (our study vs. Spanish study, 66.4 RU/ml vs. 243 RU/ml), ethnicities (Chinese vs. Spanish), and immunosuppressive regimens (cyclosporine plus corticosteroid vs. tacrolimus plus rituximab) could also contribute to the discrepancies between two studies’ results. Noticeably, Spanish study did not show the data of proteinuria and antibody titer at one-month after treatment in clinical design, which could miss information of antibody dynamic evolution at early stage. Lastly, it was important to emphasize that the relative small sample size of both studies may lack the statistical power. The specific effect of monitoring antibody titer in decision making of immunosuppressive treatment during MN patients’ follow-up was still uncertain. We need to validate the predictive thresholds of our study externally in larger population of anti-PLA2R antibody positive MN before applying them into clinical practice.

Several limitations to our study must be mentioned. Firstly, it was a non-randomized study which may produce selection bias. Secondly, some patients in CYA group were treated without monitoring trough cyclosporine level regularly, which could lead to inadequate cyclosporine dose, and consequently relative low reduction of antibody titer and proteinuria within six months after immunosuppressive treatment. Lastly, all patients were Chinese, which may limit the applicability of our finding to other ethnic populations.

## Conclusions

In conclusion, our study confirmed that serum anti-PLA2R antibody titer decline preceded the reduction of proteinuria in MN patients receiving first-line immunosuppressive medications. As for predictive value for probability of composite remission at six-month, relative reduction of anti-PLA2R antibody titer was inferior to relative reduction of 24hUP at early stage after treatment in MN patients receiving CTX daily therapy. Multi-center, prospective studies with sufficient sample size, standardized treatment and monitoring protocols would require to clarify the predictive value of anti-PLA2R antibody.

## Abbreviations

24hUP: 24-h urine protein
CKD-EPI: Chronic Kidney Disease-Epidemiology Collaboration
CR: complete remission
CTX: Cyclophosphamide
CYA: Cyclosporine A
eGFR: Estimated glomerular filtration rate
IMN: Idiopathic membranous nephropathy
MN: Membranous nephropathy
NR: No response
PLA2R: Phospholipase A2 receptor
PR: Partial remission
ROC: Receiver operating characteristic curve
SD: Standard deviation
